# Federal Investment in Primary Care Transformation: A Systematic Review and Qualitative Analysis

**DOI:** 10.1001/jamahealthforum.2025.4117

**Published:** 2025-11-07

**Authors:** Laura L. Sessums, Timothy J. Day, Lingrui Liu, Jesse C. Crosson

**Affiliations:** 1 American Board of Internal Medicine, Philadelphia, Pennsylvania; 2 Centers for Medicare & Medicaid Services, Baltimore, Maryland; 3 Agency for Healthcare Research and Quality, Rockville, Maryland

## Abstract

**Importance:**

Understanding the results of federal investment in primary care delivery and transformation is essential for informing practitioners, future program developers, and policymakers on how best to improve delivery of primary care.

**Objective:**

To identify outcomes of federal investment in primary care delivery transformation on patient experience, costs and utilization, population health, and practice experience.

**Evidence Review:**

Publicly available program evaluation reports and articles published between July 2011 (the start of the earliest identified programs) and December 31, 2024, were identified on PubMed, Scopus, CINAHL, Embase, Web of Science, and the Cochrane Library. The review included independently evaluated federal programs started after January 2011 and completed by December 2021.

**Findings:**

A total of 142 records were included in the analysis from 5 programs that met inclusion criteria: the Federally Qualified Health Center Advanced Primary Care Practice demonstration, the Multi-Payer Advanced Primary Care Practice model, the Comprehensive Primary Care (CPC) initiative, CPC Plus, and EvidenceNOW Advancing Heart Health. Programs supported practice-level changes in care delivery through payment changes, performance requirements, data feedback, and technical assistance. Federal investments were associated with substantial improvements in clinical care delivery, greater patient engagement, modest reductions in utilization, and net increases in expenditures. There was an association between practice efforts and intrinsic practice characteristics, and practices were limited by funding amounts and modality, difficulties in using electronic health records and payer data to support care improvement, staff turnover, and extrinsic factors.

**Conclusions and Relevance:**

This systematic review found that investing in primary care was associated with improvements in practice experience and population health, while outcomes regarding patient experience, costs, and utilization were mixed. Access to practice-level data and payment system challenges limited these impacts, and most outcomes were not seen until after at least 2 years. Countervailing payment incentives may have affected outcomes. Future primary care transformation efforts should focus on addressing practice-level barriers, aligning payment, and targeting support for practice-level organizational improvement based on local needs.

## Key Points

**Question:**

What are the myriad outcomes of the multifaceted federal programs to change primary care delivery from 2010 to 2021?

**Findings:**

In this systematic review of 142 records, programs supported practice-level changes in care delivery through payment changes, performance requirements, data feedback, and technical assistance, while federal investments were associated with substantial improvements in clinical care delivery, greater patient engagement, modest reductions in utilization, and net increases in expenditures. However, common measured outcomes were variable, with external factors possibly limiting impact.

**Meaning:**

Ten years of federal programs to transform primary care provides useful lessons for practices, program implementers, and policymakers.

## Teaser

This systematic review identifies outcomes of federal investment in primary care delivery transformation on patient experience, costs and utilization, population health, and practice experience.

## Introduction

When legislation was passed in 2010 (Public Law 111-148),^1^ many thought the patient-centered medical home (PCMH) and increasing inadequate primary care payment^2,3^ would improve patient experience and population health while reducing costs and improving the clinician experience, subsequently identified as the Quadruple Aim.^4,5^ Since then, private payers, states, and the federal government have invested in primary care through care delivery and payment transformation initiatives. While many of these initiatives have not been rigorously evaluated, certain federal initiatives require independent evaluation. Yet, individual publications from such initiatives are often narrowly focused and published over many years in a variety of journals and gray literature. To our knowledge, no systematic reviews have synthesized the knowledge gained from these sizable programmatic and evaluation investments.^6^

We conducted a systematic review of the major findings of independently evaluated federal initiatives in community-based primary care in the decade after enactment of the 2010 legislation. The aim was to identify the outcomes on patient experience, costs and utilization, population health, and practice experience (including organization and delivery changes).

## Methods

### Eligibility Criteria

Articles and gray literature were eligible for inclusion if they reported on federal initiatives (hereafter referred to as programs) started after January 2011 and completed by December 2021 and were aimed at enhancing community-based primary care delivery. We excluded programs in noncommunity settings, those focused on a specific population (eg, Veterans Affairs, Department of Defense, Indian Health Service), Health Resources and Services Administration health centers that are part of continuous programs (ie, not a specific, time-limited test), and accountable care organization programs (as these have a broader focus than primary care). We also excluded discussion articles, opinion pieces, editorials, clinical guidelines, trial registry records, conference abstracts, and documents focused on research design or methods. We used Endnote 20 (Clarivate) to manage this process.

We registered this systematic review protocol with PROSPERO (CRD42024564471) and report results in accordance with the relevant recommendations of the Preferred Reporting Items for Systematic Reviews and Meta-Analyses (PRISMA) guidelines.^7^ Because the review used published articles and did not collect new data or protected health information, it was exempt from institutional review board review.

### Data Sources

We developed the survey strategy with a research librarian, then searched for articles published between July 2011 (the start of the earliest identified programs) and December 31, 2024, on PubMed, Web of Science, Scopus, Embase, CINAHL, and the Cochrane Library. We searched public websites for program evaluation reports and included these in the review. See eAppendix 1 in Supplement 1 for full details.

### Record Selection

**Figure 1.**
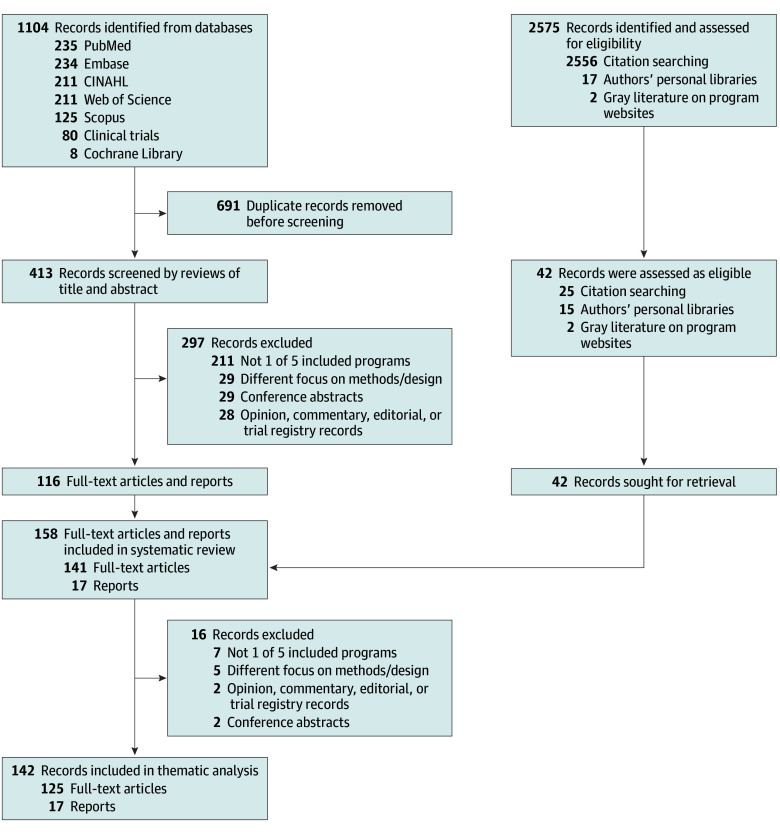
PRISMA Flowchart

Working in pairs, authors each independently screened a specific subset of titles and abstracts to determine eligibility for inclusion (disagreements resolved by consensus), then conducted full-text reviews of included records (Figure 1). Authors screened reference lists of included records to identify additional potential records. We assessed risk of bias by comparing records to evaluation plans registered on ClinicalTrials.org or from grant application specific aims.

### Data Synthesis

**Figure 2.**
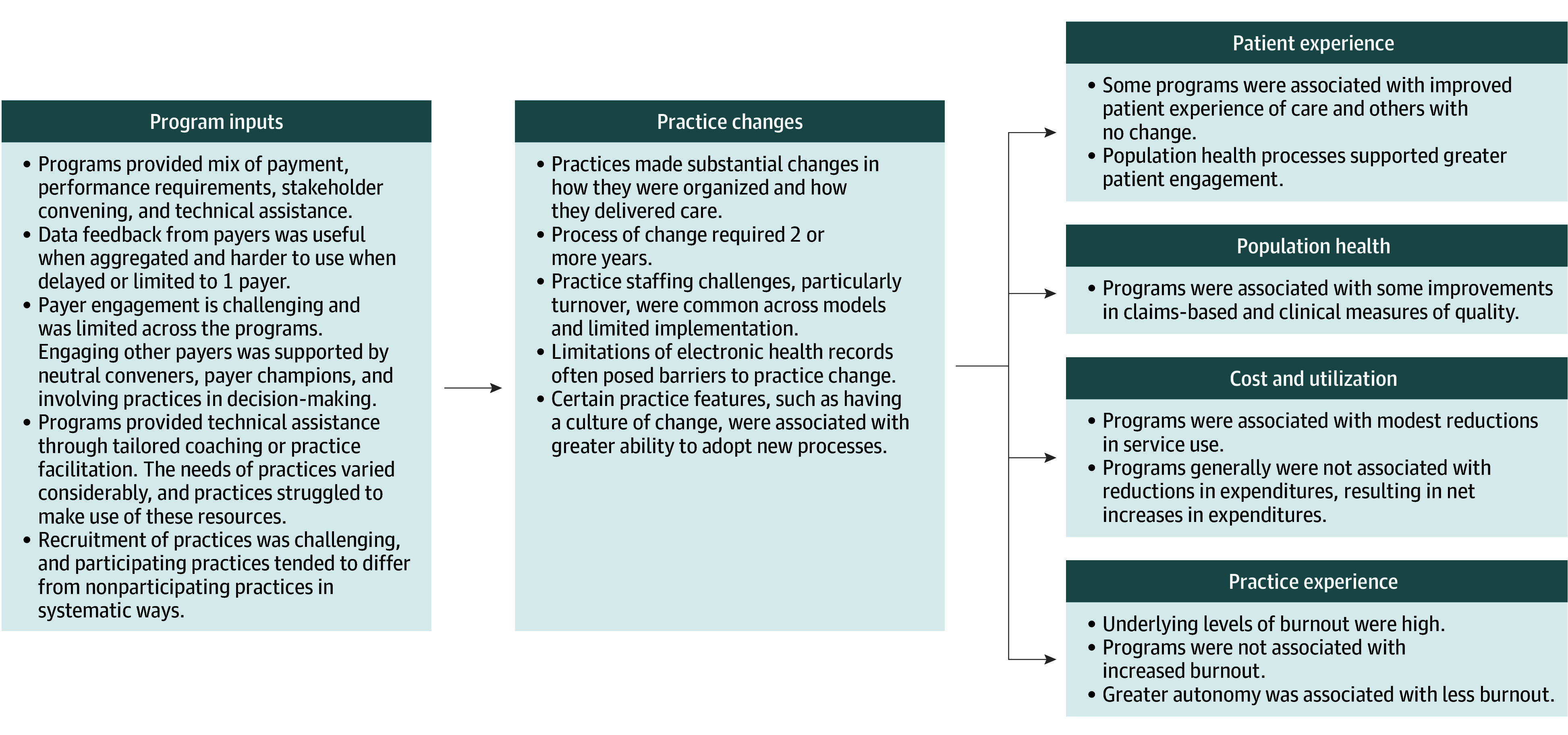
Logic Model Summary of Results

Authors reviewed the full text of each included article or report, entered key findings into a data collection form, then synthesized findings organized by program inputs and outcomes relating to patient experience, population health, cost and utilization, and practice experience (Figure 2).

## Results

### Eligible Records

Five federal programs met the inclusion criteria: the Federally Qualified Health Center (FQHC) Advanced Primary Care Practice (APCP) demonstration,^8^ the Multi-Payer Advanced Primary Care Practice (MAPCP) model,^9^ the Comprehensive Primary Care (CPC) initiative,^10^ CPC Plus (CPC+),^11^ and EvidenceNOW Advancing Heart Health (ENOW) (Table 1).^12^ The search strategy yielded 1104 records. After removing 691 duplicates, 297 records were excluded based on review of titles and abstracts (Figure 1). Reference review of the included articles and gray literature identified an additional 42 articles and reports. After further review, 16 of these were excluded, leaving 142 records for thematic analyses. Included records reported on all expected outcomes identified in the evaluation plans, indicating a low risk of publication bias.

**Table 1.** Overview of Included Community-Based Federal Primary Care Practice or Payment Transformation Models and Programs, 2011-2021

Variable	FQHC Advanced Primary Care Practice demonstration	Multi-Payer Advanced Primary Care Practice	Comprehensive Primary Care	Comprehensive Primary Care Plus	EvidenceNOW
Agency/convener	CMS	CMS/states	CMS	CMS	AHRQ
Description	CMS paid FQHCs monthly per-beneficiary CM fees to support services and activities associated with requirements for becoming an NCQA Level 3 PCMH.	CMS joined 8 state-sponsored, multipayer initiatives to promote PCMH principles among PC practices. These aspects included CM fees, medical home activity requirements, quality standards, and payment incentives.	CMS collaborated with commercial and state health insurance plans to offer population-based CM fees and shared savings opportunities to participating PC practices to support the provision of 5 PC functions: risk-stratified CM, access and continuity, planned care for chronic conditions and preventive care, patient and caregiver engagement, and coordination of care across the medical neighborhood.	Offered 2 tracks with incrementally advanced care delivery requirements and payment options. Track 1 included CM fees and incentive payments for meeting quality and utilization benchmarks. Track 2 included a hybrid payment for services that combined quarterly upfront payments with discounted per-visit payments.	Delivered support to PC practices to improve health care quality and implement evidence shown to improve heart health across 7 cooperatives. External supports included PF, health IT support, learning collaboratives, expert consultation, and data feedback and benchmarking.
Quadruple Aim objectives	Improve patient experience, population health, and practice experience; reduce costs.	Improve patient experience, population health, and practice experience; reduce costs.	Improve patient experience, population health, and practice experience; reduce costs.	Improve patient experience, population health, and practice experience; reduce costs.	Improve population health and practice experience.
Period of performance	November 2011-October 2014	July 2011-December 2016	October 2012-December 2016	January 2017-December 2021	2015-2017
Location	Nationwide	8 States	7 States or regions	18 States or regions	12 States
Participating practices	500	849	502	2905	1795
Patients/Medicare FFS beneficiaries in participating practices	Unknown/approximately 270 000	3 Million/725 000	3 Million/321 000	16.3 million/2.1 million	Approximately 8 million/unknown
Payers (includes CMS)	CMS only	4-9 Per state	39	63	NA
**Practice supports**					
Learning system	TA offered by CMS contractors and HRSA to prepare for PCMH recognition.	States offered TA for adopting the PCMH model: learning collaboratives, practice coaching, and consulting.	Practices met specified milestones. CMS supported milestone attainment with national and regional learning networks, online collaboration, and local faculty to provide hands-on assistance.	CMS provided a learning system, including detailed information on and durable resources for the PC functions and care delivery requirements, facilitated peer learning, and tailored individual practice coaching.	Cooperatives provided PF services to build local improvement capacity, TA for using health IT for quality improvement, data feedback and benchmarking, expert consultation, and learning collaboratives.
Data available to practices	Clinic readiness assessment survey scores, Medicare beneficiary cost and utilization, and beneficiary-level data for CM.	In 5 states, CMS provided practice-level quality and utilization reports based on claims data and practice-reported measures. Some states and other payers also provided data.	CMS and the majority of other payers provided claims data to practices. Five regions either aligned or aggregated claims data given to practices.	CMS provided data feedback about Medicare FFS beneficiaries through an interactive web-based tool, and 95% of payer partners individually provided data to practices about their Comprehensive Primary Care Plus patients. The payer partners in 4 regions began data aggregation efforts during Comprehensive Primary Care Plus, and in 4 additional regions payers continued existing data aggregation.	Heart health performance data from EHRs. Extent and timeliness of feedback reports varied widely across cooperatives and was dependent on local EHR functionality.
Health IT vendor support	No	No	No	CMS integrated health IT vendors into Comprehensive Primary Care Plus. These vendors committed to provide practices with the technology necessary to meet model requirements and to participate in national learning activities.	No
**Financial arrangements**					
Multipayer alignment: insurers (including Medicare, Medicaid, and commercial insurers) agree to align payment, quality measures, and data feedback; payers are convened to facilitate alignment	No	Yes^a^	Yes	Yes	No
Prospective payments for PC services: Medicare makes quarterly, upfront payments to practices for their beneficiaries based on expected PC service use	No	No	No	Track 2 only^b^	No
**CM fees**					
Average CM fees for Medicare FFS (per beneficiary, per month)^c^	$6	Up to $10; varied by state	$20 (Years 1 and 2); $15 (years 3 and 4)	Track 1, $15; track 2, $28	NA
**Financial risk or reward**					
Upside risk; shared savings: practices share in savings with Medicare if they meet specified quality-of-care targets	No	Yes^d^	Yes (performance regionally aggregated)	No	NA
Bonus payments: Medicare prospectively pays practices specified amounts (per beneficiary, per month); payments are recouped if quality targets are not met	No	No	No	Track 1, $2.50; track 2, $4	NA

Abbreviations: AHRQ, Agency for Healthcare Research and Quality; CM, care management; CMS, Centers for Medicare & Medicaid Services; EHR, electronic health record; FFS, fee for service; FQHC, Federally Qualified Health Center; HRSA, Health Resources and Services Administration; IT, information technology; NA, not applicable; NCQA, National Committee for Quality Assurance; PC, primary care; PCMH, patient-centered medical home; PF, practice facilitation; TA, technical assistance;.

^a^ Payers agreed to align on payment but not quality measurement or data feedback.

^b^ Different from CM fees; these quarterly payments are equal to a percentage of the practice’s expected Medicare payment for evaluation and management services and are combined with reduced payments on actual evaluation and management FFS claims. This hybrid approach is designed to provide flexibility to provide care outside of traditional face-to-face visits.

^c^ In the multipayer models (Multi-Payer Advanced Primary Care Practice, Comprehensive Primary Care, and Comprehensive Primary Care Plus), other payers also paid CM fees. In the Multi-Payer Advanced Primary Care Practice model, some states increased their CM fees for practices with higher NCQA medical home level recognition.

^d^ In Multi-Payer Advanced Primary Care Practice, 1 state (Pennsylvania) used the shared savings option.

### Program Inputs

The programs provided a mix of payment modalities (except ENOW), performance requirements and standards, stakeholder convening, and technical assistance resources to support practice-level organizational change and performance improvement (Table 1).^13,14,15,16^ MAPCP, CPC, and CPC+ engaged multiple payers to align practice-level activities and incentives with program requirements. Key factors associated with successful payer engagement were having a neutral convenor, support from participating payer champions, and involving practices in alignment decision-making.^17,18,19^ Except in MAPCP, where Medicare joined state-sponsored efforts,^18^ programs covered only about a third of patients in participating practices, potentially limiting the impact on practice-level care processes.^17^ The Centers for Medicare & Medicaid Services(CMS) provided the bulk of the enhanced payments to practices throughout CPC and CPC+ (70% of payments while covering 40% of patients in CPC+), and fee for service (FFS) remained the largest and most predictable revenue stream for practices.^3,17,18^

Aggregating claims data across payers for performance feedback and regional-level benchmarking proved most useful to practices for guiding change and improvement.^17,19,20,21^ The effectiveness of data feedback was limited by access barriers, claims data time lags, data quality concerns, and when data came from only from single payer.^13,15,16,17,22^ Programs relying on claims-based quality measure reports as an alternative to electronic health record–based reports required substantial investment in analytic capacity, time, ongoing technical assistance, and coordination across information technology (IT) vendors and other stakeholders.^23,24^

Technical assistance included tailored coaching or practice facilitation, providing actionable data feedback and training in using data for improvement.^3,16,17,25^ Most CMS programs used a combination of practice-specific, on-site, and peer-to-peer learning.^17,18,19^ CPC and CPC+ added detailed implementation guides.^26^ Practice facilitators supported incorporating new evidence or improvements into practice through development of practice-level capacities for quality measurement and reporting, support for work process changes, and assistance with the use of health IT to support quality improvement (QI).^3,20,27,28,29,30,31,32,33^ Practice-level barriers and needs varied widely,^34^ with health system–owned practices relying more on system-provided facilitation and technical assistance not available to independent practices.^17,35,36,37^ In ENOW, shared learning activities across practices did not yield improvement beyond practice facilitation alone.^23,24^

While practices were receptive to program supports,^15,17,35^ many still struggled to dedicate resources to local improvement efforts, with the costs of time and effort for using otherwise free support a key limiter of practice facilitation uptake.^38,39,40,41^ Practice-level disruptions may have limited practices’ ability to make QIs, although these disruptions typically did not limit participation.^42,43,44^ Given varied practice improvement support needs, facilitators required training and expertise in building relationships and trust, use of health IT, work process design, educating clinicians and staff on new evidence and processes, and the ability to tailor efforts by practice type and local concerns.^35,45,46,47,48^ In CPC+, CMS engaged electronic health record vendors to enhance functionality to support program requirements and improvement efforts, but these efforts did not fully meet practice needs.^17^

Recruitment of participants was challenging across the programs. In ENOW and MAPCP, recruitment relied on prior relationships or alignment with state-level initiatives and, in both ENOW and APCP, framing participation as aligning with other improvement efforts.^16,18,36,49,50^ Across the programs, recruited practices differed in important ways from those that did not participate.^17^ In CPC and CPC+, recruited practices tended to be in wealthier areas, with healthier and more advantaged patients, potentially missing those practices serving disadvantaged populations.^14,17,51,52^ In addition, practices that remained in CPC+ throughout the program were considerably more likely to participate in the Medicare Shared Savings Program, be owned by a hospital or health system, and have more primary care practitioners.^17^

### Program Outcomes

Changes at the practice level were associated with improvements in practice experience and population health, while associations with patient experience, costs, and utilization were mixed (Table 2). Full examination of the relationship between implementation factors and these programmatic outcomes is beyond the scope of this study. However, crosscutting and contextually meaningful implementation factors were included, and a comprehensive review of these factors is provided in eAppendix 2 in Supplement 1.

**Table 2.** Outcomes of Community-Based Federal Primary Care Practice or Payment Transformation Models and Programs, 2011-2021

Outcome	Federally Qualified Health Center Advanced Primary Care Practice demonstration	Multi-Payer Advanced Primary Care Practice	Comprehensive Primary Care	Comprehensive Primary Care Plus	EvidenceNOW
Patient experience	No association	Improved	Improved	Mixed	Not assessed
Population health	Mixed	Mixed	Improved	Improved	Improved
Cost and utilization	No association	Mixed	Mixed	Mixed	Not assessed
Practice experience	Mixed	Mixed	Mixed	Mixed	Mixed

#### Patient Experience

Although implementation varied, greater practice engagement with patients, families, and communities was seen as important for improving care delivery across most programs.^18,19,53,54,55^ Patient-reported experience improved in both CPC and MAPCP practices but not in APCP or CPC+.^17,56,57,58,59,60^ Relatedly, patient perceptions of access were not associated with the addition of care managers in CPC+.^61^ Strong relationships between patients and clinicians provided the basis for encouraging greater engagement, and building this engagement was typically easier in settings with more population health processes (such as social needs screening and using patient registries).^17,62,63^ While social needs screening increased substantially in CPC+, unavailability of community resources provided the greatest challenge to meeting identified social needs.^17,28^ Locally developed materials facilitated the engagement of patients and families in patient councils focused on practice improvement, but recruiting participants in these councils was challenging.^3,17,64,65^

#### Population Health

At the patient level, the programs led to considerable improvements in some processes and outcomes of care.

#### Processes of Care

Processes of care improved across the programs. There were improvements in use of annual eye examinations and nephropathy testing for patients with diabetes (APCP)^16,59^; chronic condition management (CPC, CPC+)^66^; continuity of care, comprehensiveness, and coordination of care (CPC, MAPCP)^3,16,17,67,68^; reduced duration (1.6%) and dose (3.5%) of long-term opioid prescriptions with overuse potential (CPC+)^69^; and smoking screening and cessation support (ENOW).^70^

#### Health Outcomes

Health outcomes improvement varied widely. Regional and practice characteristics were associated with improvements in cardiovascular disease treatment outcomes (ENOW), estimated reductions in cardiovascular events (ENOW),^40,41,46,47,71,72,73,74,75,76,77,78,79,80,81^ an estimated 1817 fewer cases of potential opioid overuse (CPC+),^69^ and no consistent change in Medicare (CPC+, MAPCP)^82^ or Medicaid health outcome measures (MAPCP).^18^ CPC showed some limited evidence that the use of electronic clinical quality measures (eCQMs) in payment systems was associated with improved patient outcomes.^83^

#### Costs and Utilization

Outcomes on emergency department (ED) visits, hospitalizations, 30-day readmissions, and costs varied across the programs. In MAPCP and APCP, overall outcomes were not meaningful.^3,18,55,59,84,85,86^ However, costs and utilization were lower in APCP practices whose patients had more regular primary care visits.^87^ In MAPCP, coordinating for transitions in care was associated with reduced ED visits, hospital admissions, and hospital spending but not utilization or expenditures for behavioral health (BH).^67,88^ Across 6 years, the CPC and CPC+ models were associated with slower growth in both Medicare hospitalizations (11 fewer per 1000 beneficiaries by year 6) and ED visits (20 fewer per 1000 beneficiaries by year 6) but not expenditures (CPC+),^82^ although costs and utilization shifted somewhat from outpatient services and skilled nursing facilities to physician services and hospice care.^17,54,66,82,89,90,91,92^ Estimates of the cross-regional effects of ENOW found reductions in direct medical costs.^57^

While outcomes in cost and utilization did not vary by practice ownership type in CPC,^92,93^ those with greater comprehensiveness were associated with reduced use of low-value services, acute care utilization, and expenditures in CPC+.^17,54,57,66,82,89,90,94,95^ Those CPC+ practices also in a Medicare accountable care organization were associated with reduced specialist visits and care costs, with these practices more frequently using referral management and adopting data-driven QI processes.^17^ In MAPCP, states that generated net savings commonly required practices to have PCMH certification, which includes an emphasis on access, continuity, and care management (CM).^96^ While practice-level performance bonuses helped to generate state-level net savings in some MAPCP states, these retrospective payments—vs stable CM fees—were associated with limited ability of practices to plan for care improvements,^18,97^ and performance incentives targeted only to primary care limited potential cost savings (CPC, CPC+).^3,17^

#### Practice Experience

Practice-level success at making program- or model-required changes varied by ownership, size, and PCMH status (ENOW, CPC, CPC+, APCP).

#### Practice Ownership

Practice ownership was a meaningful factor across multiple programs. In CPC+, system-owned practices had more implementation support, greater resources for eCQM reporting, greater access to timely patient information from hospitals, greater levels of behavioral health integration (BHI), more centralized CM resources focused on ED follow-up, and were more likely to have on-site pharmacist services.^17^ Physician-owned CPC+ practices struggled with complex participation requirements and data engagement, as they lacked data experts and QI specialists,^17^ and had higher dropout rates^17,98^; however, they were associated with reduced hospitalizations and expenditures more than system-owned practices.^17^ In ENOW, rural independent practices were more responsive to external facilitation supports,^99^ while system-owned and academic-affiliated practices implemented fewer QI strategies, experienced more burnout, had lower reported aptitude for managing change, and had less local autonomy for change and clinician engagement.^27,96,100,101,102^ However, these system-owned and academic-affiliated practices also exhibited better performance, greater use of population health approaches (such as using registries and care guidelines), and more improvement in clinical quality measures.^22,73,102,103^

#### Practice Size

Practice size was associated with the practice experience of change in ENOW, with larger practices being better at reporting eCQMs^104^ and exhibiting greater readiness for change and more improvement.^73,100^ Smaller practices demonstrated adaptability in managing change processes but faced higher burnout and dropout risks.^72,101^

#### PCMH Status

PCMH status was an important determinant of success in meeting program demands. Across CPC and CPC+, PCMH-certified (and multispecialty) practices were better able to integrate BH.^92,93^ While participation in APCP was not associated with reductions in acute care or hospitalizations, practices that achieved National Committee for Quality Assurance Level 3 PCMH recognition had better utilization, process, and spending outcomes, and APCP participation meaningfully supported achieving this recognition.^16,59^ PCMH recognition was also associated with increased use of QI for care coordination implementation, greater practice-level continuity of care, and increased likelihood of providing recommended diabetes care services and, by the end of APCP, more than 90% of the practices still participating had achieved PCMH recognition (70% at Level 3).^15,16,59,105^

#### Clinician and Staff Experience

Clinician and staff experience also varied by practice type and size. FQHCs in ENOW typically took longer to make quality improvements and reported higher burnout.^98,101,106^ Although the programs were not associated with increased underlying clinician and staff burnout, the demands of multiple overlapping improvement initiatives may have played a role.^13,101,107,108,109,110^ Practices with the highest levels of burnout were those in which clinicians had less independence or autonomy, such as health system–owned practices and FQHCs, while more facilitative practice leadership, greater psychological safety, and a learning culture within the practice were protective against burnout.^101,108,109,111,112^

## Discussion

This review of federal program evaluations provides key lessons for primary care practices, program implementers, and policymakers.

### Practices

Practices must carefully consider the care delivery changes most likely to help them achieve the Quadruple Aim, as well as the infrastructure, culture, staffing, and training required. To maximize success in transformation, practices should use their strengths and acknowledge their unique barriers. System-owned practices can take advantage of their greater resources to implement change, but they often lack practice-site autonomy, leading to a greater risk of burnout and less clinician engagement. Physician-owned practices are less burdened by hierarchy and countervailing incentives, allowing for quicker practice change, but they often lack resources for implementing complex program requirements.

Regardless of type, practices should focus on elements that facilitate practice transformation: leadership support, a practice champion, stable clinician and staff engagement, a willingness to embrace workflow and other practice changes, strong health IT functionality that supports population health management, and access and ability to use both claims and admission/discharge/transfer data to improve clinical outcomes. Note that stable and prospective payments (such as CM fees) create a dedicated revenue stream for hiring staff, while retrospective, smaller, inconsistent performance payments do not. Practices with more patients who have medically or socially complex needs may require additional supports to participate.

CM (longitudinal for a proportion of highest-risk patients and episodic for transitions of care) can benefit patients and busy physicians alike. BHI leads to increased patient BH access and adherence, better communication, and collaboration with BH clinicians, and allows for care of other patient problems. Pharmacist engagement benefits patients and helps spread workload. Social determinants of health screening is useful for prioritized needs (transportation, food insecurity, and safety topped the list)—especially if supported by health IT—though linkage to sufficient community resources is not always possible. Aside from telehealth, offering alternatives to office visit care is difficult without non-FFS payment across a practice population.

### Program Implementers

At the outset, recruitment can be difficult, particularly reaching small, independent practices and those in underserved communities. Sufficient payment provided in a manner designed to support program requirements is necessary both for initial recruitment and for ongoing practice engagement and success. Research to date has not revealed the right amount of payment. Primary care practices often perceive moving from FFS as risky, so the hybrid or capitated payment structure may be as important as the payment amounts.

In addition to payment, practices require other supports to facilitate transformation. Practices value and need learning support, including a clear road map for transformation to assist with organization and prioritization of work. Data are a powerful resource but must be accompanied by teaching data-driven QI. Orienting program requirements toward clinically valuable activities engages practices in the work. Codesign of implementation strategies by implementation scientists and clinicians may be useful.

Lack of engagement of payers for the practice population risks a misalignment between payment and requirements to change practice-wide processes and can result in practice dropout. Although not necessarily so individually, payer quality and utilization programs were motivating in the aggregate. Roadblocks to multipayer engagement include differing time horizons (longer for government payers, shorter for commercial payers), barriers to working across payers in program design, local competition between payers, payer focus on other initiatives, internal barriers to changing payment or quality measurement for program participants, and lack of leadership buy-in. An effective neutral convener for payers facilitates payer alignment.

### Policymakers

Federal, state, and local policies can affect the success of primary care practice transformation. Long-standing underinvestment in primary care means that new policies and programs must simultaneously try to right size primary care investment to meet current care demands, while sufficiently equipping and training practices to implement new care processes that can achieve the Quadruple Aim.

Multipayer engagement and alignment are essential to engage practices in transformation. Yet, the levers for engagement and alignment at the federal level are limited. States have the potential to foster engagement and alignment between payers on quality measurement, data sharing (through data aggregation or other methods), and learning support for primary care transformation. In addition, states could work toward payment parity among payers, avoiding the imbalance in CPC+ of Medicare contributing almost 70% of the enhanced payments and only 40% of the patients to the model.

Relatedly, having the right data is necessary to manage patients across the medical neighborhood (eg, referral coordination, CM during transitions, reducing unnecessary utilization). Health IT is necessary, but numerous challenges currently remain. Although Medicare engagement of vendors in CPC+ was not a panacea, government at all levels—as well as vendors—have a role to play to achieve interoperability and primary care access to timely, prioritized, useable, and useful patient data.

Given experience to date with primary care transformation, policymakers must acknowledge that Quadruple Aim improvements take time and may emerge unevenly across the aims. Expecting organizations that are paid single-digit percentages of the total cost of patients’ health care to change the cost of care is a proposition that has not borne fruit. Results from the past 10 years show that primary care transformation can reduce ED utilization and acute medical hospitalizations but not the more costly acute surgical hospitalizations. Primary care can also improve health care quality. Yet, the constant waves of FFS incentives across the health system—including FFS in the practices themselves—overwhelm the efforts to change payment to shore up the foundational health system primary care bulwark. A focus on moving away from FFS outside of primary care and making realistic goals for primary care transformation is needed. Furthermore, simplicity in payment aligned with clinical understanding may allow clinicians to run practices more easily without the need to hire business experts or forego independent practice for the safe harbor of joining a health system.

### Limitations

This review has several limitations. Its time frame omitted more recent programs, and its focus omitted other programs, such as those at the US Department of Veterans Affairs. The diversity of outcomes made a formal meta-analysis infeasible. While the underlying studies generally used rigorous designs, they are limited in their breadth and scope, limiting generalizability of findings. Spillover effects might have negatively affected the measurement of program effects on population health, cost, and utilization.^18,113^

## Conclusions

This systematic review of federal investment in primary care highlights key lessons beyond top-line effects on medical expenditures. Program benefits are clear: increases in needed services (CM, BHI, hospice), care that physicians and practices think is high quality, and reductions in acute care utilization. To deliver additional improvements and achieve success on the Quadruple Aim, the lessons of the past decade should inform the future of practice transformation efforts.

## Notes

Supplement 1. **eAppendix 1.** Search Strategy and Search Terms **eAppendix 2.** Practice-Level Implementation Factors
eReferences

Supplement 2.
Data Sharing Statement
