# Supplementary material for: Federal Investment in Primary Care Transformation: A Systematic Review and Qualitative Analysis
Source: JAMA Health Forum. 2025 Nov 7;6(11):e254117. doi: 10.1001/jamahealthforum.2025.4117 (PMC12595538; doi:10.1001/jamahealthforum.2025.4117)
Supplement: Supplement 1. — eAppendix 1. Search Strategy and Search Terms eAppendix 2. Practice-Level Implementation Factors eReferences [file jamahealthforum-e254117-s001.pdf]

Supplemental Online Content

Sessums LL, Day TJ, Liu L, Crosson JC. Federal investment in primary care transformation: a systematic review and qualitative analysis. *JAMA Health Forum*. 2025;6(11):e254117. doi:10.1001/jamahealthforum.2025.4117

eAppendix 1. Search Strategy and Search Terms

eAppendix 2. Practice-Level Implementation Factors

eReferences

This supplemental material has been provided by the authors to give readers additional information about their work.

© 2025 Sessums LL et al. *JAMA Health Forum*.

eAppendix 1. Search Strategy and Search Terms

Overall, searches combined terms from two categories: the five identified primary care transformation programs identified above and general search terms that referred to the subject matter topics, such as primary care model(s), advanced primary care, and multi-payer. The name of the relevant federal programs had to appear in qualifying articles in their entirety (e.g., “Comprehensive Primary Care (CPC)”, not “comprehensive” + “primary” + “care”). Detailed search terms and strategy are outlined as following.

PubMed

EvidenceNow OR "advancing heart health"[tiab:~3] OR “healthy hearts heartland”[tiab:~3] OR HealthyHearts OR ((“healthy heart” OR “healthy hearts”) AND “primary care”) OR “heart health now”[tiab:~3] OR “healthy hearts NYC”[tiab:~3] OR "healthy hearts northwest"[tiab:~3] OR "healthy hearts Oklahoma"[tiab:~3] OR "heart Virginia healthcare"[tiab:~3] OR “heart Virginia health care”[tiab:~3]
~~~~~~
~~~~

"comprehensive primary care initiative"[tiab:~1] OR "comprehensive primary care initiatives"[tiab:~1] OR "comprehensive primary care model"[tiab:~1] OR "comprehensive primary care models"[tiab:~1] OR "comprehensive primary care CPC"[tiab:~1] OR "comprehensive primary care plus"[tiab] OR “CPC initiative”[tiab:~0] OR "CPC plus”[tiab:~0]
~~~~~~
~~~~

"Multi Payer Advanced Primary Care Practice"[tiab:~2] OR "Multipayer Advanced Primary Care Practice"[tiab:~2] OR ("advanced primary care"[tiab] AND "multi payer"[tiab]) OR ("advanced primary care"[tiab] AND multipayer[tiab])
~~~~~~
~~~~

("federally Qualified Health Center"[tiab:~0] OR "federally Qualified Health Centers"[tiab:~0] OR FQHC[tiab]) AND (demonstration[tiab] OR demonstrations[tiab] OR “advanced primary care”[tiab] OR “advanced care”[tiab] OR “primary care model”[tiab:~3] OR “primary care models”[tiab:~3])
Filters: from 2011 – 2024

"Advanced Care Primary Practice Demonstration"[tiab:~2] Filters: from 2011 – 2024

© 2025 Sessums LL et al. *JAMA Health Forum*.

~~~~~~
~~~~

Web of Science

TS=(EvidenceNow OR "advancing heart health" OR “healthy hearts in the heartland” OR HealthyHearts OR ((“healthy heart” OR "healthy hearts") AND "primary care") OR “heart health now” OR “healthy hearts NYC” OR “healthy hearts northwest” OR “healthy hearts for Oklahoma” OR “heart of Virginia Healthcare” OR “Heart of Virginia Health Care”)

FT=(EvidenceNow OR "advancing heart health" OR “healthy hearts in the heartland” OR HealthyHearts OR ((“healthy heart” OR "healthy hearts") AND "primary care") OR “heart health now” OR “healthy hearts NYC” OR “healthy hearts northwest” OR “healthy hearts for Oklahoma” OR “heart of Virginia Healthcare” OR “Heart of Virginia Health Care”)
~~~~~~
~~~~

TS= ("comprehensive primary care initiative" OR "comprehensive primary care initiatives" OR "comprehensive primary care model" OR "comprehensive primary care models" OR "comprehensive primary care CPC" OR "comprehensive primary care plus" OR "CPC initiative" OR "CPC plus")

FT= ("comprehensive primary care initiative" OR "comprehensive primary care initiatives" OR "comprehensive primary care model" OR "comprehensive primary care models" OR "comprehensive primary care CPC" OR "comprehensive primary care plus" OR "CPC initiative" OR "CPC plus")
~~~~~~
~~~~

TS= ("Multi Payer Advanced Primary Care Practice" OR "Multipayer Advanced Primary Care Practice" OR ("advanced primary care" AND "multi payer") OR ("advanced primary care" AND "multipayer"))

FT= ("Multi Payer Advanced Primary Care Practice" OR "Multipayer Advanced Primary Care Practice" OR ("advanced primary care" AND "multi payer") OR ("advanced primary care" AND "multipayer"))
~~~~~~
~~~~

TS = (("federally Qualified Health Center" OR "federally Qualified Health Centers" OR FQHC) AND (demonstration OR demonstrations OR "advanced primary care" OR APCP OR "advanced care” OR “primary care model” OR “primary care models”))

FT = (("federally Qualified Health Center" OR "federally Qualified Health Centers" OR FQHC) AND (demonstration OR demonstrations OR "advanced primary care" OR APCP OR "advanced care” OR “primary care model” OR “primary care models”))

© 2025 Sessums LL et al. *JAMA Health Forum*.

~~~~~~
~~~~

Scopus

TITLE-ABS-KEY (EvidenceNow OR "advancing heart health" OR “healthy hearts in the heartland” OR HealthyHearts OR ((“healthy heart” OR "healthy hearts") AND "primary care") OR “heart health now” OR “healthy hearts NYC” OR “healthy hearts northwest” OR “healthy hearts for Oklahoma” OR “heart of Virginia Healthcare” OR “Heart of Virginia Health Care”)

FUND-ALL (EvidenceNow OR "advancing heart health" OR “healthy hearts in the heartland” OR HealthyHearts OR “heart health now” OR “healthy hearts NYC” OR “healthy hearts northwest” OR “healthy hearts for Oklahoma” OR “heart of Virginia Healthcare” OR “Heart of Virginia Health Care”)
~~~~~~
~~~~

TITLE-ABS-KEY ("comprehensive primary care initiative" OR "comprehensive primary care initiatives" OR "comprehensive primary care model" OR "comprehensive primary care models" OR "comprehensive primary care CPC" OR "comprehensive primary care plus" OR "CPC initiative" OR "CPC plus")

FUND-ALL ("comprehensive primary care initiative" OR "comprehensive primary care initiatives" OR "comprehensive primary care model" OR "comprehensive primary care models" OR "comprehensive primary care CPC" OR "comprehensive primary care plus" OR "CPC initiative" OR "CPC plus")
~~~~~~
~~~~

TITLE-ABS-KEY ("Multi Payer Advanced Primary Care Practice" OR "Multipayer Advanced Primary Care Practice" OR ("advanced primary care" AND "multi payer") OR ("advanced primary care" AND "multipayer"))

FUND-ALL ("Multi Payer Advanced Primary Care Practice" OR "Multipayer Advanced Primary Care Practice" OR ("advanced primary care" AND "multi payer") OR ("advanced primary care" AND "multipayer"))
~~~~~~
~~~~

TITLE-ABS-KEY (("federally Qualified Health Center" OR "federally Qualified Health Centers" OR FQHC) AND (demonstration OR demonstrations OR "advanced primary care" OR APCP OR "advanced care” OR “primary care model” OR “primary care models”))

© 2025 Sessums LL et al. *JAMA Health Forum*.

FUND-ALL (("federally Qualified Health Center" OR "federally Qualified Health Centers" OR FQHC) AND (demonstration OR demonstrations OR "advanced primary care" OR APCP OR "advanced care” OR “primary care model” OR “primary care models”))
~~~~~~
~~~~

Embase

EvidenceNow:ti,ab,kw OR "advancing heart health":ti,ab,kw OR "healthy hearts in the heartland":ti,ab,kw OR HealthyHearts:ti,ab,kw OR (("healthy heart":ti,ab,kw OR "healthy hearts":ti,ab,kw) AND "primary care":ti,ab,kw) OR "heart health now":ti,ab,kw OR "healthy hearts NYC":ti,ab,kw OR "healthy hearts northwest":ti,ab,kw OR "healthy hearts for Oklahoma":ti,ab,kw OR "heart of Virginia healthcare":ti,ab,kw OR "heart of Virginia health care":ti,ab,kw
~~~~~~
~~~~

"comprehensive primary care initiative":ti,ab,kw OR "comprehensive primary care initiatives":ti,ab,kw OR "comprehensive primary care model":ti,ab,kw OR "comprehensive primary care models":ti,ab,kw OR "comprehensive primary care CPC":ti,ab,kw OR "comprehensive primary care plus":ti,ab,kw OR "CPC initiative":ti,ab,kw OR "CPC plus":ti,ab,kw
~~~~~~
~~~~

"Multi Payer Advanced Primary Care Practice":ti,ab,kw OR "Multipayer Advanced Primary Care Practice":ti,ab,kw OR ("advanced primary care":ti,ab,kw AND "multi payer":ti,ab,kw) OR ("advanced primary care":ti,ab,kw AND multipayer:ti,ab,kw)
~~~~~~
~~~~

("federally Qualified Health Center":ti,ab,kw OR "federally Qualified Health Centers":ti,ab,kw OR FQHC:ti,ab,kw) AND (demonstration:ti,ab,kw OR demonstrations:ti,ab,kw OR "advanced primary care":ti,ab,kw OR APCP:ti,ab,kw OR "advanced care”:ti,ab,kw OR “primary care model”:ti,ab,kw OR “primary care models”:ti,ab,kw)
~~~~~~
~~~~

CINAHL

EvidenceNow OR "advancing heart health" OR “healthy hearts in the heartland” OR HealthyHearts OR ((“healthy heart” OR "healthy hearts") AND "primary care") OR “heart health now” OR “healthy hearts NYC” OR “healthy hearts northwest” OR “healthy hearts for Oklahoma” OR “heart of Virginia Healthcare” OR “Heart of Virginia Health Care”

GI (EvidenceNow OR "advancing heart health" OR “healthy hearts in the heartland” OR HealthyHearts OR “heart health now” OR “healthy hearts NYC” OR “healthy hearts northwest” OR “healthy hearts for Oklahoma” OR “heart of Virginia Healthcare” OR “Heart of Virginia Health Care”)

© 2025 Sessums LL et al. *JAMA Health Forum*.

~~~~~~
~~~~

"comprehensive primary care initiative" OR "comprehensive primary care initiatives" OR "comprehensive primary care model" OR "comprehensive primary care models" OR "comprehensive primary care CPC" OR "comprehensive primary care plus" OR "CPC initiative" OR "CPC plus"

GI ("comprehensive primary care initiative" OR "comprehensive primary care initiatives" OR "comprehensive primary care model" OR "comprehensive primary care models" OR "comprehensive primary care CPC" OR "comprehensive primary care plus" OR "CPC initiative" OR "CPC plus")
~~~~~~
~~~~

"Multi Payer Advanced Primary Care Practice" OR "Multipayer Advanced Primary Care Practice" OR ("advanced primary care" AND "multi payer") OR ("advanced primary care" AND "multipayer")

“Advanced Primary Care Practice”

GI ("Multi Payer Advanced Primary Care Practice" OR "Multipayer Advanced Primary Care Practice")

GI (“Advanced Primary Care Practice”)
~~~~~~
~~~~

("federally Qualified Health Center" OR "federally Qualified Health Centers" OR FQHC) AND (demonstration OR demonstrations OR "advanced primary care" OR APCP OR "advanced care” OR “primary care model” OR “primary care models”)

GI (("federally Qualified Health Center" OR "federally Qualified Health Centers" OR FQHC) AND (demonstration OR demonstrations OR "advanced primary care" OR APCP OR "advanced care” OR “primary care model” OR “primary care models”))

“Advanced Primary Care Practice Demonstration” OR “Advanced Primary Care Practice (APCP) Demonstration”

GI “Advanced Primary Care Practice Demonstration” OR “Advanced Primary Care Practice (APCP) Demonstration”
~~~~~~
~~~~

Cochrane Library

All text (EvidenceNow OR "advancing heart health" OR "healthy hearts heartland" OR HealthyHearts OR (("healthy heart" OR "healthy hearts") AND "primary care") OR “heart health

© 2025 Sessums LL et al. *JAMA Health Forum*.

now” OR "healthy hearts NYC" OR "healthy hearts northwest" OR "healthy hearts Oklahoma" OR "heart Virginia healthcare" OR "heart Virginia health care")
~~~~~~

All text ((comprehensive near/5 "primary care" near/5 (initiative* OR model*)) OR "comprehensive primary care" OR "CPC initiative" OR "CPC plus")
~~~~~~

All text ("Multi Payer Advanced Primary Care Practice" OR "Multipayer Advanced Primary Care Practice" OR ("advanced primary care" AND ("multi payer" OR “multi payers” OR multipayer OR multipayers)))
~~~~~~

All text (("federally Qualified Health Center" OR "federally Qualified Health Centers" OR FQHC) AND (demonstration* OR “advanced primary care” OR APCP OR “advanced care” OR (“primary care” near/5 model*)))
~~~~~~

eAppendix 2. Practice-Level Implementation Factors

Practice inner setting

In all programs, participating practices made substantial changes in their organization for and delivery of care. Longstanding underinvestment in primary care meant that changes required two or more years to manifest in improved infrastructure and new processes and to gain leadership support for continued improvement.[1-6] Most primary care practices across both ENOW and CPC+ had insufficient staffing for implementing new models of care and had limited ability to dedicate staff to improvement efforts.[2,7,8] Staffing challenges were common across programs with shortages, turnover, and other disruptions negatively affecting QI efforts.[2,6,7,9,10] Staff turnover was particularly disruptive as it commonly increased workloads on remaining staff and limited retention of institutional knowledge.[6,10,11] Practices able to dedicate protected time for staff to work on QI were more likely to be high performing and better able to engage with external supports.[12-14]

The EHR capabilities available to primary care practices proved to be important structural features for both effective participation in QI and, ultimately, to making quality improvements.[11,15-17] Common technical limitations of EHRs included poor interoperability with other health information technology (health IT) systems, inability to generate actionable reports for QI, challenges extracting data or generating quality measure reports suitable for QI work, and regular updates and changes in EHR systems.[2,6,7,9,18-21] These limitations reduced the ability of practices to fully engage with QI processes, and practices with greater ability to extract actionable data from their EHR more easily implemented quality improvements.[5,22,23]

© 2025 Sessums LL et al. *JAMA Health Forum*.

Practice-level factors associated with greater ability to adopt new processes and requirements included having a culture of change, higher levels of organizational capacity for change, leadership support, previous QI experience, and an organizational climate favoring learning and psychological safety.[1,6,11,16,24-27] Experience from these programs showed that clinician and staff engagement, leadership engagement (both clinical and managerial), tailoring interventions to local needs while maintaining key programmatic elements, and having a receptive organizational culture aligned with programmatic objectives were important drivers of implementation success.[1,3,4,27-31]

CPC+ generated widespread satisfaction and high practice retention, and practice staff and other clinicians expressed satisfaction with the ability to use enhanced payment to support greater access to care and improved CM relative to FFS.[32,33] Participants in CPC/CPC+ and APCP sought to expand the definition of a practice from the physical, bricks-and-mortar definition used by CMS to a broader practice definition (such as tax identification number) that reflects the organizational-level budgeting and staffing decisions commonly made in these settings.[4,6] In addition, practices saw reporting requirements (especially the financial reporting) as overly burdensome.[4]

Innovation characteristics

In APCP, practices used care management fees to support hiring CM staff, EHR modifications, and training activities in support of PCMH certification.[6] In MAPCP, practices increased access via multiple modalities, though the addition of care coordinators was considered most transformative and shared support teams were highly

© 2025 Sessums LL et al. *JAMA Health Forum*.

valued.[5] In CPC and CPC+, practices used enhanced payments to add care managers and QI staff and to expand the availability of behavioral health (BH) services within the practice.[1,4,34,35] CPC+ practitioners increased by 11% by the end of the program.[4] The addition of these new staff members enhanced access to care and provided both longitudinal CM, leading to improved chronic condition care and episodic CM.[1,4] However, challenges with developing the CM workforce and concerns about the usefulness of automatic risk stratification rather than clinician judgment were barriers to more widespread acceptance of these efforts.[4,36-38]

Most CPC+ payers did not move away from FFS and practices were hesitant to forgo FFS for additional Medicare non-FFS payments (viewing this as additional capitation risk), resulting in reluctance to embrace alternatives to visit-based care not covered by FFS.[4] The CPC+ program requirements for comprehensive medication management led to a marked increase in the integration of pharmacists into practices (increasing from 19% to 54%).[4] Furthermore, requirements for BH integration (BHI) led to an increase in availability of these services, despite workforce shortages.[4,35]

© 2025 Sessums LL et al. *JAMA Health Forum*.

eReferences

1. Peikes D, Taylor EF, O'malley AS, Rich EC. The changing landscape of primary care: Effects of the ACA and other efforts over the past decade. Review. *Health Affairs*. 2020;39(3):421-428. doi:10.1377/hlthaff.2019.01430
2. Meyers D, Miller T, De La Mare J, et al. What AHRQ Learned While Working to Transform Primary Care. *Annals of Family Medicine*. Mar-Apr 2024;22(2):161-166. doi:10.1370/afm.3090
3. Goetz Goldberg D, Haghighat S, Kavalloor S, Nichols LM. A Qualitative Analysis of Implementing EvidenceNOW to Improve Cardiovascular Care. *Journal of the American Board of Family Medicine*. Sep-Oct 2019;32(5):705-714. doi:10.3122/jabfm.2019.05.190084
4. Mathematic Policy Research. Independent Evaluation of Comprehensive Primary Care Plus (CPC+): Final Report (2023). https://www.mathematica.org/publications/independent-evaluation-of-comprehensive-primary-care-plus-cpc-final-report
5. Nichols D, Haber S, Romaire M. Evaluation of the Multi-Payer Advanced Primary Care Practice (MAPCP) Demonstration Final Report (2017). https://downloads.cms.gov/files/cmmi/mapcp-finalevalrpt.pdf
6. Kahn KL, Timbie JW, Friedberg MW, Mendel P, Hiatt L, Chen EK. Evaluation of CMS's Federally Qualified Health Center (FQHC) Advanced Primary Care Practice (APCP) Demonstration: final report (2017). https://downloads.cms.gov/files/cmmi/fqhc-finalevalrpt.pdf
7. Ye J, Zhang R, Bannon JE, et al. Identifying Practice Facilitation Delays and Barriers in Primary Care Quality Improvement. *Journal of the American Board of Family Medicine*. Sep-Oct 2020;33(5):655-664. doi:10.3122/jabfm.2020.05.200058
8. Peikes DN, Reid RJ, Day TJ, et al. Staffing patterns of primary care practices in the comprehensive primary care initiative. *Annals of Family Medicine*. Mar-Apr 2014;12(2):142-9. doi:10.1370/afm.1626
9. McHugh M, Heinrich J, Philbin S, et al. Declining Participation in Primary Care Quality Improvement Research: A Qualitative Study. *Annals of Family Medicine*. Sep-Oct 2023;21(5):388-394. doi:10.1370/afm.3007
10. Baron AA-O, Hemler JR, Sweeney SA-O, et al. Effects of Practice Turnover on Primary Care Quality Improvement Implementation. *Am J Med Qual*. (1555-824X (Electronic))doi:10.1177/1062860619844001
11. Nguyen AM, Cuthel AM, Rogers ES, et al. Attributes of High-Performing Small Practices in a Guideline Implementation: A Multiple-Case Study. *Journal of Primary Care & Community Health*. Jan-Dec 2020;11:2150132720984411. doi:10.1177/2150132720984411
12. Soylu TG, Cuellar AE, Goldberg DG, Kuzel AJ. Readiness and Implementation of Quality Improvement Strategies Among Small- and Medium-Sized Primary Care Practices: an Observational Study. *Journal of General Internal Medicine*. Oct 2020;35(10):2882-2888. doi:10.1007/s11606-020-05978-w
13. Roberts MM, Marino M, Wells R, Atem FD, Balasubramanian BA. Differences in Use of Clinical Decision Support Tools and Implementation of Aspirin, Blood Pressure Control, Cholesterol Management, and Smoking Cessation Quality Metrics in Small Practices by Race and Sex. *JAMA Network Open*. Aug 2023;6(8):e2326905. doi:10.1001/jamanetworkopen.2023.26905
14. Edwards ST, Marino M, Balasubramanian BA, et al. Burnout Among Physicians, Advanced Practice Clinicians and Staff in Smaller Primary Care Practices. Article. *Journal of General Internal Medicine*. 2018;33(12):2138-2146. doi:10.1007/s11606-018-4679-0
15. Lavelle TA, Rose AA-O, Timbie JW, et al. Utilization of health care services among Medicare beneficiaries who visit federally qualified health centers. *BMC Health Serv Res*. (1472-6963 (Electronic))

© 2025 Sessums LL et al. *JAMA Health Forum*.

16. Petersen DM, O'Malley AS, Felland L, et al. Reducing Acute Hospitalizations at High-Performing CPC+ Primary Care Practice Sites: Strategies, Activities, and Facilitators. *Annals of Family Medicine*. Jul-Aug 2023;21(4):313-321. doi:10.1370/afm.2992
17. LaBonte CT, Payne P, Rollow W, et al. Performance on Electronic Clinical Quality Measures in the Comprehensive Primary Care Initiative. *American Journal of Medical Quality*. Mar/Apr 2019;34(2):119-126. doi:10.1177/1062860618794868
18. Parchman ML, Baldwin LM, Howell R, Hummel J. The Ability of Primary Care Practices to Measure and Report on Care Quality. *Journal of the American Board of Family Medicine*. Mar-Apr 2024;37(2):316-320. doi:10.3122/jabfm.2023.230116R1
19. Liss Dt Fau - Peprah YA, Peprah Ya Fau - Brown T, Brown T Fau - Ciolino JD, et al. Using Electronic Health Records to Measure Quality Improvement Efforts: Findings from a Large Practice Facilitation Initiative. *Jt Comm J Qual Patient Saf*. (1938-131X (Electronic))doi:10.1016/j.jcjq.2019.09.006
20. Cohen DJ, Dorr DA, Knierim K, et al. Primary Care Practices' Abilities And Challenges In Using Electronic Health Record Data For Quality Improvement. *Health Affairs*. Apr 2018;37(4):635-643. doi:10.1377/hlthaff.2017.1254
21. Nichols D, Haber S, Romaire M. Evaluation of the Multi-Payer Advanced Primary Care Practice (MAPCP) Demonstration: Second Annual Report (2016).
22. Balasubramanian BA, Marino M, Cohen DJ, et al. Use of Quality Improvement Strategies Among Small to Medium-Size US Primary Care Practices. *Ann Fam Med*. (1544-1717 (Electronic))doi:10.1370/afm.2172
23. Knierim KE, Hall TL, Dickinson LM, et al. Primary Care Practices' Ability to Report Electronic Clinical Quality Measures in the EvidenceNOW Southwest Initiative to Improve Heart Health. *JAMA Network Open*. Aug 2 2019;2(8):e198569. doi:10.1001/jamanetworkopen.2019.8569
24. Goldberg DG, Soylu TG, Kitsantas P, Grady VM, Elward K, Nichols LM. Burnout among Primary Care Providers and Staff: Evaluating the Association with Practice Adaptive Reserve and Individual Behaviors. *Journal of General Internal Medicine*. May 2021;36(5):1222-1228. doi:10.1007/s11606-020-06367-z
25. Goldberg DG, Owens-Jasey C, Haghighat S, Kavalloor S. Implementation strategies for large scale quality improvement initiatives in primary care settings: a qualitative assessment. *BMC Primary Care*. Nov 17 2023;24(1):242. doi:10.1186/s12875-023-02200-8
26. Schuttner L, Coleman K, Ralston J, Parchman M. The role of organizational learning and resilience for change in building quality improvement capacity in primary care. *Health Care Management Review*. Apr-Jun 2021;46(2):E1-E7. doi:10.1097/hmr.0000000000000281
27. Ross SM, Wang A, Anthony L, Persell SD, Yu J, Kho AN. Is more better? The impact of implementing more interventions for hypertension control in a practice facilitation study for small- and medium-sized practices. *Journal of Human Hypertension*. Nov 2023;37(11):1007-1014. doi:10.1038/s41371-023-00813-1
28. Nguyen AM, Cuthel A, Padgett DK, et al. How Practice Facilitation Strategies Differ by Practice Context. *Journal of General Internal Medicine*. Mar 2020;35(3):824-831. doi:10.1007/s11606-019-05350-7
29. Cohen DJ, Sweeney SM, Miller WL, et al. Improving Smoking and Blood Pressure Outcomes: The Interplay Between Operational Changes and Local Context. *Annals of Family Medicine*. May-Jun 2021;19(3):240-248. doi:10.1370/afm.2668
30. Soylu TG, Cuellar AE, Goldberg DG, Kuzel AJ. Engagement of Small to Medium-Sized Primary Care Practices in Quality Improvement Efforts. *Journal of the American Board of Family Medicine*. Jan-Feb 2021;34(1):40-48. doi:10.3122/jabfm.2021.01.200153

31. Abramsohn E, DePumpo M, Boyd K, et al. Implementation of Community-Based Resource Referrals for Cardiovascular Disease Self-Management. *Ann Fam Med*. (1544-1717 (Electronic))
32. Peikes DN, Swankoski K, Hoag SD, et al. The Effects of a Primary Care Transformation Initiative on Primary Care Physician Burnout and Workplace Experience. *Journal of General Internal Medicine*. Jan 2019;34(1):49-57. doi:10.1007/s11606-018-4545-0
33. Cohen G, Duda N, Morrison-Lee K, et al. How CPC+ supported patient care during the COVID-19 pandemic: Lessons for alternative payment models. *Healthcare*. Apr 10 2024;12(2):100745. doi:10.1016/j.hjdsi.2024.100745
34. Swankoski KE, Peikes DN, Palakal M, Duda N, Day TJ. Primary Care Practice Transformation Introduces Different Staff Roles. *Annals of Family Medicine*. May 2020;18(3):227-234. doi:10.1370/afm.2515
35. Santos T, Bergman A, Smith-McLallen A. Access to Mental Health and Substance Use Treatment in Comprehensive Primary Care Plus. *JAMA Network Open*. Apr 2024;7(4):e248519. doi:10.1001/jamanetworkopen.2024.8519
36. Higgins TC, O'Malley AS, Keith RE. Exploring and Overcoming the Challenges Primary Care Practices Face with Care Management of High-Risk Patients in CPC+: a Mixed-Methods Study. *Journal of General Internal Medicine*. Oct 2021;36(10):3008-3014. doi:10.1007/s11606-020-06528-0
37. Reddy A, Sessums L, Gupta R, et al. Risk Stratification Methods and Provision of Care Management Services in Comprehensive Primary Care Initiative Practices. *Annals of Family Medicine*. Sep 2017;15(5):451-454. doi:10.1370/afm.2124
38. O'Malley AS, Peikes D, Wilson C, et al. Patients' perspectives of care management: a qualitative study. *American Journal of Managed Care*. Nov 2017;23(11):684-689.

© 2025 Sessums LL et al. *JAMA Health Forum*.
