# Supplementary material for: Federal Investment in Primary Care Transformation: A Systematic Review and Qualitative Analysis
Source: JAMA Health Forum. 2025 Nov 7;6(11):e254117. doi: 10.1001/jamahealthforum.2025.4117 (PMC12595538; doi:10.1001/jamahealthforum.2025.4117)
Supplement: Supplement 2. — Data Sharing Statement [file jamahealthforum-e254117-s002.pdf]

Data Sharing Statement

Sessums. Federal Investment in Primary Care Transformation. *JAMA Health Forum*. Published November 07, 2025. doi:10.1001/jamahealthforum.2025.4117

Data
Data available: No

Additional Information
Explanation for why data not available: Our review uses only publicly available studies and the data are available through those sources
